# Assessing the implementation of nurse practitioner-led huddles in long-term care using the Consolidated Framework for Implementation Research (CFIR)

**DOI:** 10.1186/s12912-023-01354-1

**Published:** 2023-06-07

**Authors:** Aria Wills, Alexandra Krassikova, Margaret Keatings, Astrid Escrig-Pinol, Jennifer Bethell, Katherine S. McGilton

**Affiliations:** 1 KITE Research Institute, Toronto Rehabilitation Institute – University Health Network, 500 University Avenue, Toronto, ON Canada; 2 Bruyère Research Institute, 43 Bruyère Street, Ottawa, ON Canada; 3 ESIMar (Mar Nursing School), Parc de Salut Mar, Universitat Pompeu Fabra-affiliated, Barcelona, Spain; 4 SDHEd (Social Determinants and Health Education Research Group), IMIM (Hospital del Mar Medical Research Institute), Barcelona, Spain; 5 Institute of Health Policy, Management and Evaluation, University of Toronto, 155 College Street, Toronto, ON Canada; 6 Lawrence S Bloomberg Faculty of Nursing, University of Toronto, 155 College Street, Toronto, ON Canada

**Keywords:** Long-term care, Nursing home, Nurse practitioner, Huddles, Consolidated framework for implementation research

## Abstract

**Background:**

The COVID-19 pandemic created major challenges in long-term care (LTC) homes across Canada and globally. A nurse practitioner-led interdisciplinary huddle intervention was developed to support staff wellbeing in two LTC homes in Ontario, Canada. The objective of this study was to identify the constructs strongly influencing the process of implementation of huddles across both sites, capturing the overall barriers and facilitators and the intervention’s intrinsic properties.

**Methods:**

Nineteen participants were interviewed about their experiences, pre-, post-, and during huddle implementation. The Consolidated Framework for Implementation Research (CFIR) was used to guide data collection and analysis. CFIR rating rules and a cross-comparison analysis was used to identify differentiating factors between sites. A novel extension to the CFIR analysis process was designed to assess commonly influential factors across both sites.

**Results:**

Nineteen of twenty selected CFIR constructs were coded in interviews from both sites. Five constructs were determined to be strongly influential across both implementation sites and a detailed description is provided: evidence strength and quality; needs and resources of those served by the organization; leadership engagement; relative priority; and champions. A summary of ratings and an illustrative quote are provided for each construct.

**Conclusion:**

Successful huddles require long-term care leaders to consider their involvement, the inclusion all team members to help build relationships and foster cohesion, and the integration of nurse practitioners as full-time staff members within LTC homes to support staff and facilitate initiatives for wellbeing. This research provides an example of a novel approach using the CFIR methodology, extending its use to identify significant factors for implementation when it is not possible to compare differences in success.

**Supplementary Information:**

The online version contains supplementary material available at 10.1186/s12912-023-01354-1.

## Contributions to the literature

- Systemic issues in LTC were exacerbated by the COVID-19 pandemic, including personnel shortages, increasing resident acuity and mortality, and rising staff distress and burnout.
- Engagement of management, staff, and clinical leaders such as nurse practitioners was significant for successful implementation of a huddle intervention to support LTC staff’s wellbeing.
- These findings address existing gaps in the literature, specifically identifying the factors that may impact the process of implementation of a well-established intervention.
- This study employed the CFIR methodology in a unique approach to identify commonalities in implementation processes between two implementation sites.

## Background

The demands facing health care teams in long-term care (LTC) homes have rapidly escalated in previous decades. The number of older adults requiring full-time care continues to increase beyond available accommodations [1]. Further, the acuity of LTC residents continues to grow, with significant increases in their cognitive and physical impairments upon admission [2]. Simultaneously, the number of staff has not kept pace with care requirements and both in Canada and globally, LTC homes report difficulty in recruiting and retaining staff, resulting in shortages of direct and non-direct care staff [3, 4].

These challenges were exacerbated by the COVID-19 pandemic leading to further “catastrophic” consequences [5]. Staff were limited in their work for a multitude of new reasons, including infection with COVID-19 and restriction to single workplaces for infection prevention. LTC home staff experienced burnout [6], increasing turnover [7], and worsening mental health including post-traumatic stress and mood disturbances [8]. Taken together, rising unmet resident needs, inadequate staffing, and resulting staff distress and dissatisfaction compromised effective person-centred care, and negatively impacted quality of life for LTC residents [9, 10].

Effective communication and support amongst staff and strong organizational leadership have been shown to enable positive staff experiences, for instance, in the use of huddles [6–8, 11]. Irrespective of role, staff who participate in huddles report improved teamwork, supportive practice environments, and self-efficacy in the LTC home context [12]. Soft skills such as strong leadership, communication, and effective listening facilitates the implementation of evidence-based interventions, such as huddles, by addressing various competencies and needs of interdisciplinary groups [13].

Researchers designed an interdisciplinary huddle intervention to address staff wellbeing in two LTC homes in Ontario, Canada. Huddles were implemented during the COVID-19 pandemic. Nurse practitioners (NPs) have demonstrated the capacity for supporting staff, enhancing collaboration, and mentorship [14, 15], and thus, an NP at each LTC home was chosen as the huddle facilitator. Compared to pre-intervention, staff who participated in these huddles reported lower levels of moral distress and greater perceived support from the NP facilitator. The complete intervention design and outcomes associated with one LTC home have been submitted for publication [16].

While other studies have reported on outcomes of huddle implementation in LTC homes [12, 17, 18], optimal implementation strategies for this intervention and the structural and individual factors that impact implementation remain unclear. This study employed the Consolidated Framework for Implementation Research (CFIR) to analyze the implementation process of interdisciplinary huddles in two LTC homes. Using this framework captured the multi-level nature of implementation strategies, particularly within the evolving contexts of LTC homes [19]. The CFIR has been established as a valuable tool across healthcare settings for assessing implementation and can support content analysis of qualitative data [20, 21]. The aim of this study was to identify the CFIR constructs that strongly influenced both implementation sites, captured as overall barriers and facilitators to the implementation process and properties intrinsic to the intervention.

## Methods

### Setting

Nurse Practitioner-led interdisciplinary huddles were implemented in two LTC homes in Ontario, Canada. An email was sent through the Nurse Practitioner Association of Ontario (NPAO) seeking NPs interested in participating in a research project to address staff wellbeing via an implementation study. Two NPs self-identified to the PI (KM) and recruited the administrator of the LTC homes where they worked. Both NPs practiced within a Nurse Practitioner Led Outreach Team (NLOT), providing episodic, acute resident care.

Site 1 was a private not-for-profit LTC home with < 150 beds located in a large town. The home was divided into five units, two of which implemented the huddles for day and evening staff. Each unit accommodated approximately 32 residents, and care was provided by one registered practical nurse (RPN), personal support workers (PSWs) and resident support aides (RSAs). The RSA role was introduced during the COVID-19 pandemic to support PSWs in the provision of non-caregiving tasks. A charge registered nurse (RN) was responsible for overseeing all five units of the home. The NP associated with this home worked on a contract basis, providing a total of eight hours of care to the entire LTC home, between two to four times per week. The NP also provided support for staff, additional palliative care on weekends, and was scheduled on-call as a part of the NLOT team. The NP worked at the LTC home full-time for two months throughout the first wave of the pandemic. During the 15-week study, they carried out 48 huddles between the two units.

Site 2 was a municipal public LTC home with > 250 beds located in a medium-sized city. The home was divided into ten units and huddles were implemented concurrently on two units for staff working day and evening shifts. Each unit accommodated approximately 20 residents. Both units shared one RN, with care provided on the units by one RPN each, as well as PSWs and RSAs. The NP providing care in the home assumed on-call responsibilities as part of the NLOT team to provide acute, episodic care. This NP held a total of 12 huddles over 4 weeks. After 4 weeks the implementation was terminated prematurely; due to the changing environment of COVID-19, the NP had to attend to other responsibilities. There were no other NPs available to assume this role and continue with the intervention.

### Participants

Participants who had previously attended a workshop to develop the huddle intervention and reported attending the huddles were invited to be interviewed. In Site 1, 16 interviews were conducted with 12 individuals. Six interviews were conducted pre-implementation (3 management, 1 direct care provider, 1 non-direct care provider, and 1 NP), and 10 were conducted post-implementation (5 management, 4 direct care providers, and 1 NP). Four individuals (3 management, 1 NP) were interviewed at both time points.

In Site 2, 10 interviews were conducted with 9 individuals (2 management, 5 direct care providers, 1 non-direct care provider, 1 NP) throughout the implementation process. One manager was interviewed twice.

The characteristics of participants from Sites 1 and 2 are summarized in Table 1.

**Table 1:** Interview participant characteristics (*N* = 21)

Characteristic	Site 1 (*n* = 12)	Site 2 (*n* = 9)
Age (Years)		
18–34	1	0
35–44	2	3
45–54	7	6
55 <	2	0
Gender		
Women	11 (92%)	8 (89%)
Men	1 (7%)	1 (11%)
Ethnicity		
White	10 (91%)	8 (89%)
Non-White	1 (9%)	1 (11%)
Role Type		
*Management*		
CEO	1	0
Administrator	1	1
Director of Care	1	1
Assistant Director of Care	1	0
Quality & Risk Management Lead	1	0
Nurse Practitioner	1	1
*Direct Care Providers*		
RN	1	1
RPN	3	1
PSW	1	3
*Non-Direct Care Providers*		
Infection Prevention and Control Specialist	0	1
Recreation Facilitator	1	0
Role Experience (Years)		
< 1	1	1
1–5	3	4
6–15	5	3
16 <	2	1

### Intervention

A multidisciplinary huddle intervention was developed to address staff concerns and improve staff wellbeing with the ultimate goal of improving resident care. All disciplines on participating units were invited to attend including direct care staff (RNs, RPNs, PSWs, RSAs), non-direct care staff (dietary, recreation, housekeeping staff) and organizational management. It was initially implemented and facilitated by one NP in each LTC home, with the aim of transitioning the facilitator role to a staff member on the unit.

### Data Collection

Semi-structured, qualitative interviews were undertaken by telephone, by two research coordinators (RCs) (AK [MSc], AW [BSc]) and one principal investigator (KM [PhD]). Informed written consent was granted beforehand electronically. The study protocol was approved by the Toronto Rehabilitation Institute, University Health Network Research Ethics Board (REB#20-6298). A semi-structured interview guide was developed using the CFIR to prompt participants to reflect on their experience throughout the implementation process (see Supplementary Appendix A) [22, 23]. Interviews lasted approximately 35 min and were audio recorded, then transcribed verbatim and anonymized by AW.

### Data Analysis

#### Coding

Twenty CFIR constructs of 39 total were selected a priori by AK as codes that were considered relevant to the interview questions and potential influences for the setting, intervention, and participants. All transcripts were then coded separately using NVIVO [24] by RCs (AK, AW). All lines were assigned a CFIR construct code using the constructs’ definition adapted for this intervention based on the CFIR codebook where possible, and for the rest, new codes were generated by RCs. Each transcript was reviewed together by RCs and an additional analyst (MK), and discrepancies were discussed to reach consensus.

#### Rating constructs

Codes from each site were aggregated into memos in Microsoft Word, using an adapted version of the CFIR template [22]. Pre- and post-implementation interviews from Site 1 were aggregated separately into two memos. Interviews from Site 2 occurred throughout the shortened course of implementation, and were aggregated in one memo, resulting in a total of three memos. Memos were arranged first by CFIR construct and then grouped by participant. Ratings were assigned by RCs separately using the rating rules provided by CFIR [22]. This entailed assessing the construct for valence (+/-) and strength (0, 1, 2). Asterisks (*) were used to indicate mixed findings in valence within participants’ comments. Ratings were assigned to each interviewee within a construct, as an aggregate representation of all statements collected. The individual valence ratings were then aggregated to produce an overall rating for each construct. Overall ratings that were mixed were marked as ‘X’. Each RC also composed a summary of the construct elaborating on their reasoning and findings supporting their ratings. This was performed for each construct within each site. RCs met to compare, discuss discrepancies, and reach consensus for each construct and summary.

### Analysis and Interpretation

Analysis of CFIR constructs was managed in Microsoft Excel. In line with conventional use of the CFIR, the study team (KM, AK, AW, MK) identified constructs as ‘Distinguishing’ factors to identify differences in the implementation process between sites. Because implementation was terminated prematurely at Site 2, timepoints were not synchronous between sites. Based on their respective implementation timelines, Site 1’s post-implementation overall ratings were compared to Site 2’s overall ratings for greatest accuracy in comparison. The team labelled each construct as ‘Strongly Distinguishing’, ‘Weakly Distinguishing’, or ‘Not Distinguishing’ based on a construct’s dominance, i.e., constructs with the greatest discordance in valence and strength between sites, those with greatest reported frequency, and those that appeared to have distinguished the sites based on researchers’ judgement. This identification process was performed individually by the team and disagreements were discussed to reach consensus. Distinguishing constructs are not expanded upon in our results.

A conventional CFIR comparison identifies distinguishing factors in order to explain differences in implementation success [25]. Actual differences in success could not be defined in this study, as implementation was prematurely terminated due to extenuating circumstances and not intrinsic to the implementation process. An additional analysis process was thus created to identify commonalities in the process across both sites, to capture the remaining significant findings and those factors described as influential to implementation by participants. Constructs labelled as ‘Not Distinguishing’ between sites were assessed as ‘Influential factors’ across sites. Constructs were labelled as ‘Strongly Influential’, ‘Weakly Influential’, or ‘Not Influential’ based on the strength of the sites’ ratings, and the nature of participants’ comments. The complete process is described in Supplementary Appendix B.

## Results

Nineteen of 20 selected CFIR constructs were coded in interviews from both sites. 10 constructs were determined to be similarly influential across sites (5 weakly influential, 5 strongly influential). Strongly influential constructs (evidence strength and quality; needs and resources of those served by the organization; leadership engagement; relative priority; and champions) are expanded upon in our results. Table 2 provides a rating summary and an illustrative quote for each construct.

**Table 2:** Ratings assigned to CFIR construct by Site

	LTC Home 1 Rating	Illustrative Quote	LTC Home 2 Rating	Illustrative Quote	Distinguishing Factor between sites	Influential Factor across sites
**I. INTERVENTION CHARACTERISTICS**						
Evidence Strength & Quality	+ 2	“I think the staff really find them beneficial. It gives them an opportunity to talk and brainstorm together as a team to come up with interventions … It helps to talk about it and know that we are listening.” (03)	+ 2	“[Huddles] really are probably one of the stronger solutions of building a strong team and fostering a compassionate workplace. And ultimately, what that benefits is the care that we provide at the frontline. And that’s what we want.” (14)		**
Relative Advantage	+ 2	“Shift report is only [going] through what happens to residents, their health status, what we should delegate and pay attention to. Huddles are more like solving, brainstorming. Sometimes it’s just one little problem on the unit and it causes everyone trouble … Once we eliminate that, everybody has a better day.” (20)	+ 1	“There was no actual formal process to [previous debriefing]. They maybe recognize that we’re doing it in an informal way, but I don’t think they recognize the importance of doing it formally, and doing it consistently, and how impactful it really truly can be.” (14)	*	
Complexity	2	“It wasn’t this big meeting that people had to stop their work and interrupt their work plan – it was something that we could do short-term for them.” (01)	+ 1*	“I don’t think I would see it [as complex]. I think it was a very logical way of supporting the staff and practical way of supporting staff during a very stressful time, to have that opportunity for staff to be heard.” (10)		*
Design Quality & Packaging	+ 2	“The [materials] were useful. We used the [whiteboards] to put down ideas or put down questions. And sometimes when a huddle is done, when I get back to the unit there is a new question or a new suggestion on it, and I’m like, “Okay, well that’s good.” It gives people a place to put down their ideas or their questions. So, it was good. The materials provided were good.” (07)	+ 1	“That was really helpful. I think that really supported the work and supported the intervention, so I wasn’t having to make up themes and topics, etcetera. And the tools for feedback and completing that feedback, those were incredibly helpful. Really helped to be able to consolidate the information and put notes. I think I would have been lost without those tools.” (09)	*	
**II. OUTER SETTING**						
Needs & Resources of Those Served by the Organization	- 2	“Everybody is working hard, and they have a list of things to do, so can they or do they have time to stop doing that and add another [thing] on the plate? So, that’s the challenge. So, I think that the willingness is there, it’s just the logistics of finding [participants]” (05)	- 2	“Again, we’re going back to not having enough staff… If I had a fourth person, I could add five minutes onto the morning report. You see? Because those five minutes will get lost in a way, because there is more staff, and it will get done much faster.” (17)		**
**III. INNER SETTING**						
Communication	+ 2*	“I found coming to this home, I thought, ‘Boy, they really communicate well.‘ So, outbreaks updates; the administrator did a full report, that was sent out regularly; they communicated point-click-care; they have a Facebook group. So, there were multiple forms of communications available.” (06)	- 2*	“[Leadership] will send out this email… But the majority of the PSWs don’t read their emails. They don’t have time during their shift to read their emails. RPNs read their emails because they’re doing charting, they’re on their computer; PSWs aren’t… It’s come up multiple times at meetings, “What can we do to get this message out to staff?” And we’re all drawing blanks.” (12)	**	
Culture	- 1	“[Staff] just need to come forward and ask, and they need to have confidence to know that they can do that, they’re allowed. And I think that we have an open enough organization that that can be instilled… We need to work on that one for sure.” (02)	+ 1*	“[Leadership] wants staff to connect. We have the idea of staff connection. To connect, they need to know what’s happening, and what happened yesterday… And I think [staff] like that, anything that will improve the work for us.” (16)	*	
Implementation Climate: 1. Tension for Change	+ 1*	“Well, I think if we could do it now, this year like we did, in the state we’re in, I think that shows a lot. Because hopefully things are going to get better and it’s going to get much easier to fit into the day-to-day.” (03)	- 2	“I think they see it as a pain in the butt, ‘What is the value in this?’ And that’s always the challenge with anything new that you want to implement – it’s the buy-in… And so, that is a barrier to begin this.“ (14)	**	
Implementation Climate: 2. Compatibility	+ 2*	“Absolutely [huddles fit within the home]. I think they fit in any long-term care home to be honest with you… Because often they’re nursing-focused. So having the whole team – the dietary, the program activity people, the housekeepers, the nursing teams – that’s really effective. Because then they are part of the team.” (04)	- 2*	“I don’t know if there would ever be a right moment to do it, that’s for sure. So, I can see some struggle in implementing it, but I’m not sure that waiting is necessary and would make it better.” (11)	**	
Implementation Climate: 3. Relative Priority	- 2*	“And it’s just that struggle to meet the basic care needs. There’s a lot of quality improvement things that can be introduced but it’s introducing them properly that they’re going to work well, right? And when you’re just trying to make the basic needs everyday it kind of stops you in your tracks from what you want to do.” (19)	- 2	“For every minute you take to do something else, you’re taking away from care that could be provided to residents. So, every minute that you’re in a meeting, every minute that you’re in a huddle today is a minute that you’re not participating in taking care of somebody. And so, there’s an opportunity lost there, when you’re having a huddle.” (12)		**
Implementation Climate: 4. Organizational Incentives & Rewards	Missing		- 1	“Staff are not really happy or maybe not really fully engaged [with huddles] because of the tasks that they need to complete. And we’re going to be questioning them as leaders or management if they don’t complete their tasks.” (16)		
Readiness for Implementation: 1. Leadership Engagement	- 2*	“I’m concerned about the nursing management team, because to me they should be one of the people that to continue to lead the huddles going forth. And I’m not sure if they’re ready – not even ready, but hopefully they will be able to keep the sustainability of it.” (04)	- 2	“I think it would have been helpful to have presence of management and leadership at these huddles… Even if there was an opportunity moving forward, that management were at so many huddles, or once a month, or whatever that looks like. Or if there were certain triggers – a patient safety issue, etcetera – what would trigger management to then come into these huddles? Because I think their presence really is important” (09)		**
**IV. CHARACTERISTICS OF INDIVIDUALS**						
Knowledge & Beliefs about the Innovation	+ 2*	“I haven’t heard anything negative about it. I haven’t heard any negative feedback… I do think, just in general, most staff are receptive to anything that’s going to help them in the long run. I think we have a pretty good team here, and they do overall work well together. So hopefully with the extra support, that will help further.” (06)	+ 2*	“I think it was a very logical way of supporting the staff and practical way of supporting staff during a very stressful time, to have that opportunity for staff to be heard… I think they can help to keep relationships strong amongst the teams. Because ultimately, I do believe the stronger the teams, the better the care and the better the work experience and living experience for residents.” (10)	*	
Self-Efficacy	- 2*	“It felt like [staff] were happy with nurse practitioner to be asking questions and to be leading how the meeting goes and answering and giving ideas. Rather than taking that role and start asking everybody… I think some people don’t want to be doing anything other than their position at work, like PSWs and other people.” (20)	0	“I did try to encourage the staff, “Now that you’ve seen what we’ve done, you guys can all do this on your own. If you have a concern about a resident, think about how you could pull together your own little group and do that.” And I don’t know if that continues.” (09)	*	
Individual Identification with Organization	- 2*	“I don’t want to say PSWs are the lower rung, but we feel that way sometimes – one of the upper ones got to hear and see, “Oh. Well, that’s a simple fix.” Then they got to jump over to a different department, and they got to fix it. Instead of the other department going, “Well, I’m not changing that on a PSW’s say.” (21)	0	“And sometimes, I talk to the girls, “As long as we do our job, they’re not going to give us more staff.” But you see, our souls cannot not finish the job because this is why we are there. We are there because we want to help people.” (17)		*
Other Personal Attributes	+ 2*	“The nurse practitioner is a good leader… because she’s very positive, she’s very knowledgeable and she actually gets things done… And she listens. We feel that she listens.” (21)	- 1	“During an outbreak or in the midst of the pandemic, I can see how that would be very helpful. I think post-pandemic, it’s maybe a little bit different. You’re talking about different things. And I think some of the topics that we wanted staff to talk about require personal relationships with their team and with the person who is leading the huddle.” (10)	**	
**V. PROCESS**						
Engagement	+ 1*	“It’s probably going to be 60:40 with 60 [percent of staff] being the positive… Like I said, everybody has different problems and different personalities. I think for the most part, it’s going to help. Going to give a 60:40, but it all depends too on who can facilitate the huddles for those other 40% strong personalities.” (05)	+ 1*	“I think some [staff are receptive to huddles], yes, some of them not. It’s something new – some people don’t want because they like to work by themselves. Some people are more together. If you work together, it’s much easier.” (15)		*
Engagement: 1. Opinion Leaders	- 2	“The recreational therapy manager… her group are a very important part of the team… I pulled her into one huddle… [Instead of engaging,] she felt like she needed to explain why they do the work they do, why they’re not available all the time, that they have to do charting too.” (01)	0	“I honestly believe that a huddle is only going to work on a floor where you’ve got supervisor buy-in for it. If you don’t have a team lead or an RN who is buying into it and making time for it, it’s not going to happen on the floor.” (12)		*
Engagement: 2. Champions	- 1*	“Some people don’t want to step out and start leading the huddles. The environment of the group on the unit is usually that the RPN is the lead and PSWs, they usually do not. And they always say, ‘That’s why we don’t want to be a nurse, because we don’t want the responsibility’… Nobody really wants to step out of their role and do something else.” (20)	- 2*	“But I (NP) did ask them to look to [continue the huddles] and I tried to build the capacity like, ‘You don’t need me here to do these huddles, you guys can do these independently. Think of what it would look, think of how I’ve done these.’ So, I did work hard to build that capacity and that independence so that they could do these huddles outside of just having me present… And I don’t know if that continues.” (09)		**
Engagement: 3. Innovation Participants	+ 2*	“So, it’s really effective to have from your dietary to your housekeeper to resident support aides all contributing towards the [same] goal… When they’re a part of that huddle, that they’re all members of the team. And I believe in and always say that we all need each other to function as a unit.” (04)	X	“I think some of them [are engaged], yes, some of them not. It’s something new – some people don’t want because they like to work by themselves. Some people are more together.” (15)		*

### "An opportunity to speak & be heard"

*Evidence strength and quality* was rated consistently positively across sites, participants perceived the intervention as effective and believed that it would address staff needs. Huddles were regarded as an opportunity for staff to communicate more effectively with each other and with leadership. Participants noted that staff were able to discuss topics they may not have usually addressed with leadership due to time constraints or lack of comfort in approaching management. Staff described feeling heard in the huddles and were able to discuss topics which leadership. In the words of an NP, “[I] may not be able to do anything about it, but just recognizing that they have concerns” (01 NP) *Given the sample size, we do not provide further information on participants, such as their site, to prevent identification and preserve their anonymity. In addition, participants described having greater and timelier access to information. As a direct care provider (DCP) noted, they accessed information they would not have “on a good day” (07 DCP). Huddles also provided an opportunity for interdisciplinary problem solving amongst staff regarding both staff wellbeing and resident care, as a manager explained:

“Just being able to communicate as a multi-disciplinary team and take down those silos – the housekeeping, the nursing department, the different departments all kind of having struggles over the same things.” (19 Management)

Huddles also provided the opportunity for connection between disciplines. Staff described the intervention as effective in team building. An NP said that huddles promoted “team comradery … and support for the teams within” (09 NP). One DCP noted that huddles allowed participating individuals to realize that they were all members of the same interdisciplinary team (07 DCP). Furthermore, staff believed their wellbeing and morale was improved. A DCP explained that “when staff know each other on a personal level and they understand what struggles people are going through, they’re more willing to understand and support each other” (14 DCP).

Developing solutions during the huddles also gave staff some peace of mind. One DCP reflected that they were “not just sitting and being angry and upset and tired. We’re actually trying to improve something, change something” (20 DCP). Staff worked with the NP to create action and follow-up plans and develop opportunities for improvement within the home. By improving communication, connection, and problem-solving, participants agreed that the intervention’s strengths were beneficial to the home and effective in ultimately supporting quality of care of care provided. One DCP recognized that “ultimately, what [huddles] benefit is the care that we provide at the frontline” (14 DCP).

### "Time and stress are the issue"

The *needs and resources* of staff were rated as strongly negative across both sites; capacity to address staff needs was lacking. Prior to implementation and emerging from the second wave of the COVID-19 pandemic, participants described care providers as experiencing an “overall burnout” (01 NP). The LTC homes struggled to maintain adequate staffing, one DCP noting they were “often working short or doing more than one role at the same time” (06 DCP). Management reported spending much of their time finding available staff, and one site ultimately hired agency staff, a solution that managers described as a last resort. Chronic short staffing posed a challenge to huddle implementation as staff found it difficult to participate and respond to care needs simultaneously:

“Staff are pulled from the time that they are supposed to be finishing their charting, answering the call bell, putting the residents on the toilet – and they are taken from that time. And they’re sitting there, they’re really worried – they’re not even fully focused because they need to go back and finish their job.” (16 DCP)

The possibility of having just one additional person working on the unit was viewed by participants as potentially beneficial in addressing this concern. They perceived that this would allow staff to focus on the huddles, since the extra support would make up any time taken away from direct care. One DCP explained, “in a perfect world, you replace me [on the floor] and then we can have a huddle, no problem” (17 DCP). In Site 1, such a novel role was described as a formalized position of resident coordinator, a role that would ensure continuity and communication surrounding the huddles. In Site 2, rather than creating a new role, they suggested introducing an additional staff member “to watch the call bell or to actually pick up what [DCPs] are supposed to be completing” (16 DCP). However, difficulty filling these positions in both sites meant staff were not provided with this resource. Because of this, staff were not always able to participate in the huddles. NPs worked to accommodate staff’s schedules, to find a more available time of day, such as during shift handover.

### Leaders “running on quicksand”

The *engagement of leadership* in both sites was viewed as strongly negatively influential. Leaders voiced their commitment to the intervention but did not have the capacity for optimal involvement and accountability, particularly as implementation took place during the COVID-19 pandemic. Participants agreed across sites that there was buy-in from leadership, who were described as passionate and receptive. As described by a DCP, “they support anything that ultimately would benefit the care that we provide to the residents” (14 DCP). This commitment from leadership was evident to the researchers based on their receptivity to and support in introducing the research intervention.

However, participants believed that due to conflicting responsibilities this intervention had become a lower priority for leadership, reducing their participation. Management expressed difficulties in “getting away from their desks … We can try as hard as we like but it’s not easy” (02 Management). They described specific competing responsibilities, including visits from the Ministry of Long-Term Care, resolving resident critical incidences, and addressing the staffing crisis. One manager stated, “the last two days I’ve done nothing but scheduling and really my heart’s out on the units” (19 Management). Leadership agreed that they would like to take on a role in supporting the huddles, however, as one manager explained, “I just haven’t been able to attend them as much as I wanted to or be as much of a support as I hoped to be … We were just running on quicksand and just trying from this end to help as much as we can” (19 Management). Like staff, management were described as experiencing burnout. As one NP described, “managers are distressed and unless you can really support the managers and help them move forward, how can they support the staff?” (01 NP). Managers agreed that this was exacerbated by the COVID-19 pandemic. One manager stated it became “incredibly challenging to manage anything during this pandemic beyond everyday operations” (10 Management).

All participants spoke of the value of leadership involvement to ensure consistent and sustainable huddles. Staff voiced the importance of leaders as role models in “organizing and setting aside the time” (13 Non-direct care provider [NDCP]) to huddle, and the authority of top-level managers to say, “[huddles] are what we’re doing. And we’re sticking with it.” (04 Management). Participants suggested that optimal leadership engagement would entail attendance at huddles, particularly when discussing topics pertaining to their roles and responsibilities. For instance, a DCP noted management’s presence would be important “if management is part of solving the problem, either dealing with the family or resident or something drastic that has been happening on the unit” (20 DCP). The NP and DCPs described difficulties in following through to resolve particular issues raised during the huddle when it exceeded their role capacity. One DCP noted topics such as “questions about staffing or … stuff that’s out of [a DCP’s] control” (22 DCP). One NP described the additional workload they took on trying to “mitigate some of [communication issues] amongst the home and the staff” (09 NP) that are more readily resolved with the managers’ participation in huddles.

Participants perceived an leadership engagement as a priority. One DCP stated, “I don’t know that they recognize or understand how important their actual participation in it is too” (14 DCP). NPs and staff emphasized the importance for leadership presence at huddles. One NP explained, “I think the staff really want to know that their concerns, or even the good things that they do are heard by the people that lead them” (01 NP). This would allow management to interact and engage with staff. The other NP agreed, stating managers could “have their ear to the ground so to speak … Then they’re there on the frontlines, they’re seeing what the staff are seeing, they’re hearing their concerns directly.” (09 NP). They perceived benefits beyond improving staff morale, participants suggested leadership engagement would contribute to staff participation in the intervention. One DCP said, “I think to get the buy-in of the majority of the staff, we’re going to have to see senior management be willing to be a part of these huddles” (12 DCP). However, some participants noted that when management were able to attend, they observed a shift in the dynamic of communication, in that “people were not as open” or “free to talk” (07 DCP). It was suggested that this would be resolved when “[huddles] become more like a routine thing” (20 DCP), thus allowing more time for staff to acclimate to leadership presence and continue to engage effectively in the huddles.

### “A delicate balance” between staff support & resident care

The *relative priority* of the intervention was perceived as low amongst all participants, thus strongly, negatively influencing implementation at both sites. Participants acknowledged the intervention’s importance, however, agreed that with limited capacity at both sites resident care was prioritized:

“Everyone you pull someone away from the floor, you’re actually taking them from the residents, which means residents receive less support. There’s always that delicate balance of trying to ensure that we provide as much care as humanly possible to the residents to ensure their overall support and safety but also ensure that teams are supported.” (09 NP).

One manager described care staff as “struggling to meet the basic care needs … which kind of stops you in your tracks from what you want to [implement]” (19 Management). Participants identified the staffing crisis as the cause of this difficulty. Both sites described their teams as “chronically short-staffed” (18 Management) and noted that this would remain a challenge for all LTC homes, particularly emerging from the COVID-19 pandemic. One manager stated, “everyone’s in shortage. People are walking away from long-term care” (04 Management). As a result, increases in workload meant it was “difficult to actually allocate that time” to the intervention, one manager emphasized that “everyone’s role is so so stretched” (11 Management). Participants described staff experiencing greater stress due to time spent in the huddles. One manager noted, “even if it’s just 15 minutes, it’s enough to derail their day” (10 Management). Participants described it as difficult to identify willing facilitators for the huddles, as “nobody really has the capacity right now” (11 Management).

Over time, however, participants perceived that the intervention increased in priority, suggesting that the length of the implementation period may be important for determining its success. Despite the time commitment, huddles were perceived to ultimately improve staff’s capacity for resident care as huddles provided solutions to daily challenges. Participants agreed that a decrease in huddle frequency to once or twice weekly would allow for more effective changes to be enacted, as, unlike acute care where huddles originated, the LTC home setting does not change rapidly day-to-day: “So we actually have things that move and change and then … if we’re having a problem, get together and talk about it” (20 DCP).

### "Who is going to take charge?"

The *champions* for the huddles were those who were most dedicated to leading implementation and overcoming resistance. Participant comments were mixed regarding this construct. As an NP-led intervention, the NP role was essential for initiating and sustaining early implementation. Subsequently, the champion role was designed to be transitioned to staff at the unit, however, was met with resistance. One manager noted, “The best of intentions [for implementation] would flounder without having leadership” (02 Management). This construct was overall strongly, negatively influential for the intervention’s sustained implementation.

As the initial huddle facilitators, both NPs were passionate and committed to the intervention. One manager explained that the NP’s “level of engagement and enthusiasm and passion for [their] work contributed to good outcomes” (10 Management). The multifaceted role of the NP was credited with success as facilitator, as they leveraged their skills as clinicians and leaders to build staff’s clinical skill, but also provide emotional support:

“That’s part of the [NP] role, is leadership … We’re able to talk about clinical practice, we’re able to talk about de-stressing and debriefing which is sort of our counselling role … we have that connection, we go and talk to the families, we assess the resident, we can build skills.” (01 NP).

Participants believed it was beneficial that the initial champions driving the intervention were third parties with good knowledge of residents. One NP suggested, “I think they were more comfortable sharing and talking with me and sharing their concerns” (09 NP). With regards to clinical issues, one manager noted, “the NP gives confidence that [staff] have a well-informed, well-educated person” (02 Management). Additionally, the NPs’ position of authority in both sites meant they “actually get things done” (21 DCP) and could persevere in resolving issues involving interdisciplinary collaboration and management involvement. Both NPs noted the challenges associated with this role; additional time to ensure effective implementation, including encouraging participation, engagement, and sustainability, while also communicating with leadership and facilitating remote huddles when required all increased their workload.

NPs encouraged unit leaders to sustain the huddles, “I did work hard to build that capacity and that independence so that they could do these huddles outside of just having me present” (09 NP) noted one NP. However, this transition did not occur “smoothly … because they were used to the nurse practitioner” (04 NDCP) and the staff were reluctant to assume the role of champion. One participant suggested, “Maybe it’s because we had such a hard year and everybody’s tired and overworked. So, they weren’t really willing to jump in and fill in the position” (20 DCP). Unit leaders agreed that they “don’t want that responsibility because [they] have enough responsibilities” (07 DCP). In comparison to the NP role, staff felt unequipped. One participant described their hesitancy in dealing with “all those questions and having to come up with ideas or brainstorming or going to find someone to answer the questions” (07 DCP). Leaders on the unit expressed concern that facilitating and addressing the concerns raised during the huddles were beyond their scope of knowledge and power. Staff suggested that a more formal process with ongoing support might help them prepare for this transition. This construct was revealed to be significant in implementation sustainability, as participants agreed it was unclear who would continue leading and sustaining the huddles, without the NP as champion.

## Discussion

This study employed the CFIR in a novel approach to provide insights into similar factors influencing the process across sites. Due to incomplete implementation of the intervention in Site 2, direct comparison of each site’s success was not possible, and identifying differences in the process was not feasible through conventional analysis. This unique approach allowed for examination of common influential factors across both implementation sites. This novel approach will be beneficial for future implementation science projects regardless of the level of completion as it will provide insights into the implementation process for projects that are terminated prematurely. Using the CFIR, five constructs were found to strongly influence the implementation of interdisciplinary NP-led huddles across two LTC homes. For successful huddles to occur, LTC leaders must consider their involvement in huddles, the importance of including all team members in the huddles to help build relationships and foster cohesion, and integrate NPs as full-time staff members within the home.

Short-staffing and excessive workload was a challenge cited by participants of all disciplines, impacting intervention implementation and staff’s daily responsibilities. Addressing these concerns would reduce the potential for missed resident care. However, solutions lie beyond simply adding resources, as increasing the number of direct care and allied staff alone does not reduce missed care [26]. Other factors, including negative job satisfaction and higher rates of burnout, predicted greater care rationing and missed care [27, 28]. Strong interdisciplinary teamwork has been shown to address many of these challenges; improving group cohesion creates a satisfying work environment, lessens burnout, reduces staff turnover, and leads to less missed care [29, 30]. Participants in this study asserted a strong positive *belief in the intervention* and agreed that *evidence* supported the effectiveness of interdisciplinary huddles to support staff wellbeing. Staff perceived breaking down divisions between disciplines very positively and appreciated the feeling of working together as members of the same team. Building and enhancing relationships amongst staff at all levels and across all disciplines, for instance using an interdisciplinary huddle intervention, provides one strong solution to staffing and wellbeing challenges.

The findings of this study suggest that managers would benefit from enhanced relationships with staff, and this could be done by listening to and addressing staffs’ concerns. Staff consistently voiced the need for their leadership team to take part in the intervention. However, management in both LTC homes described their aim to actively participate in huddles but a diminished capacity to do so because of conflicting responsibilities. Leadership plays an important role in effectively identifying and utilizing resources [31] and managers reported addressing *needs and resources* of the home as a chief priority. Leaders underscored time spent scheduling and locating available staff, with one home ultimately hiring agency staff and working closely with the local nursing college to address staffing shortages. However, managers’ perceptions of the work environment tend to differ significantly from staff’s perceptions [32]. This disconnect could be addressed with communication directly from leadership, for instance, in the setting of a huddle where staff concerns could be heard and resolved.

Management attendance may further facilitate the implementation process by demonstrating empowering support behaviour, which can increase staff’s confidence and self-perceived abilities [33]. Staff whose negative self-assessment as *champions* may be bolstered from this support and feel confident to take on this role. The hesitancy of staff to take up the huddle facilitator role as *champions* highlights issues of resourcing new roles as opposed to empowering current staff. RNs have been shown to improve nursing outcomes beyond the addition of other care professionals [34]. However, since 2013, the number of RNs working in LTC has decreased while the proportion of RPNs and PSWs has continued to increase [3]. The RN role aligns with that described by participants as resident coordinator to maintain huddle sustainability and accountability. Charge nurses must prioritize their own responsibilities, coordinate staff at the unit level in their daily tasks, and monitor resident care for quality [35]. RNs have demonstrated competence when given additional responsibilities, such as staff empowerment through coaching [36], suggesting the role of charge nurse is ideally situated to improve organization at the direct care level, and may be ideal facilitators.

The findings of this study also elucidated the need to reconsider the NPs’ integration in LTC organizations. The *personal attributes* of each NP regarding the differing amount of time each spent in the LTC home affected their capacity to build and maintain strong rapport with staff and a consistent presence in the home. As nursing leaders, NPs role of building staff capacity requires relationships and group cohesion [35, 37]; these differences in the NP function and responsibility within an organization may have ultimately impacted the success of the intervention. Despite one NP’s capacity to spend more time in the LTC home, both were ultimately characterized as external contract staff. NP facilitators holding “full-time, permanent” roles could optimize their capacity for rapport-building, flexibility and mentoring to benefit future huddle implementation and staff facilitation.

Two main limitations to this study need to be acknowledged. First, this study was conducted with two NPs in two LTC homes, therefore, findings might not be representative of the broader experiences of NPs in LTC homes. However, the rigorous use of the CFIR framework to underscore commonalities and variances strengthened the study and enhanced its relevance. Second, data were collected from staff through interviews, however, other perspectives such as residents and care partners were not included. While multiple staff roles were included in the study, allowing us to deepen our understanding of implementation, future research could examine residents’ and care partners’ perspectives.

## Conclusion

There is an urgent need to identify strategies to improve wellbeing of staff and leaders within LTC homes. Successful implementation of one such initiative requires the engagement of leaders, staff, and change champions, such as NPs, who are in a prime position to facilitate such initiatives. As LTC homes emerge from the wake of the COVID-19 pandemic, evolving leadership strategies, creative staffing solutions, and novel models of care are being considered. Future research is needed to deepen our understanding of how different care provider configurations may influence implementation processes, and quality, resident, and staff outcomes.

## Supplementary Information

**Additional file 1: Supplementary Appendix A.** Semi-Structured Interview Guide. Site 1 Pre-Implementation & Site 2 Interview Guide.

**Additional file 2: Supplementary Appendix B. **Phases of CFIR Analysis. Description of process of thematic analysis.

## Data Availability

The datasets generated and/or analysed during the current study are not publicly available due participant confidentiality but are available from the corresponding author on reasonable request.

## Abbreviations

CFIR: Consolidated Framework for Implementation Research
DCP: Direct Care Provider
LTC: Long-Term Care
NDCP: Non-Direct Care Provider
NLOT: Nurse Practitioner Led Outreach Team
NP: Nurse Practitioner
NPAO: Nurse Practitioner Association of Ontario
PSW: Personal Support Worker
RC: Research Coordinator
RN: Registered Nurse
RPN: Registered Practical Nurse
RSA: Resident Support Aide
