# Supplementary material for: Assessing the implementation of nurse practitioner-led huddles in long-term care using the Consolidated Framework for Implementation Research (CFIR)
Source: BMC Nurs. 2023 Jun 7;22:193. doi: 10.1186/s12912-023-01354-1 (PMC10245368; doi:10.1186/s12912-023-01354-1)
Supplement: Supplementary file 1 — Additional file 1: Supplementary Appendix A. Semi-Structured Interview Guide. Site 1 Pre-Implementation & Site 2 Interview Guide. [file 12912_2023_1354_MOESM1_ESM.docx]

***Supplementary Appendix A.* Semi-structured Interview Guide**

*Site 1 Pre-Implementation & Site 2 Interview Guide*

1. What was your experience with the long-term care home throughout the COVID-19 pandemic?

2. During the pandemic, did you have any concerns with the care delivered by the home?

3. Did you observe any challenges encountered by the workforce (nurses, personal support workers) through the pandemic?

4. Can you describe any actions carried out by the home in response to the workforce challenges?

5. In your opinion, how do you think these challenges can be prevented in the future?

6. Is the home ready to implementation the huddles intervention?

7. What do you think of the complexity of the intervention?

8. In your opinion, do you think staff are receptive of it?

9. Is there adequate communication between LTCH members about the intervention?

10. How can staff receive the support from the NP and administrators they need to implement the intervention?

11. Do the LTCH policies support the implementation of the intervention?

12. Does the intervention fit within the home?

*Site 1 Post-Implementation Interview Guide*

1. What is your experience as a participant in the huddles?
2. What worked well with the huddles intervention?
3. What did not work well with the huddles?
4. In your opinion, what makes a good facilitator?
5. In your opinion, were the staff receptive of the huddles?
6. Can you describe how the huddles changed over time, between leaders, within or between units, etc.?

*(Probe: Differences from shift report, topics discussed, manager presence?)*

1. In your opinion, how was the transition of leadership in the intervention between facilitators?
2. If you were asked to make a recommendation to improve the huddles, what would you recommend?
3. What did you think about the complexity of the huddles?
4. Was the home ready to implement the intervention?
5. Did staff receive the support they needed to implement the intervention from the NP and administrators?
6. Ultimately, did the intervention fit within the home/unit/your role?
7. Do you intend to continue using the huddles in the future?
