# Supplementary material for: Assessing the implementation of nurse practitioner-led huddles in long-term care using the Consolidated Framework for Implementation Research (CFIR)
Source: BMC Nurs. 2023 Jun 7;22:193. doi: 10.1186/s12912-023-01354-1 (PMC10245368; doi:10.1186/s12912-023-01354-1)
Supplement: Supplementary file 2 — Additional file 2: Supplementary Appendix B. Phases of CFIR Analysis. Description of process of thematic analysis. [file 12912_2023_1354_MOESM2_ESM.docx]

***Supplementary Appendix B.* Phases of CFIR Analysis (adapted from Damschroder et al., 2009)**

| **Phases of thematic analysis** | **Description of the process** |
| --- | --- |
| 1. Familiarization with the data | 1.1. The research coordinators (RCs [AW, AK]) each conducted interviews via phone, with the other RC acting as notetaker on the line.  1.2. After each interview, the RCs debriefed and discussed emerging topics.  1.3. The full corpus of interviews was transcribed by AW. |
| 2. Construct coding | 2.1. Prior to beginning the analysis in NVIVO12 the primary analyst (AK) generated a list of 20 CFIR constructs selected from the 39 total in the framework, based on the interview guide and initial themes identified by the RCs and NTs in step 1.2:  *Domain*: Innovation Characteristics; *Constructs*: Evidence strength and quality, Relative advantage, Complexity, Design quality and packaging.  *Domain*: Outer Setting; *Construct*: Needs and resources of those served by the organization.  *Domain*: Inner Setting; *Constructs*: Networks and communication, Culture, Tension for change, Compatibility, Relative priority, Organizational incentives and rewards, Leadership engagement.  *Domain*: Characteristics of Individuals; *Constructs*: Knowledge and beliefs about the intervention, Self-efficacy, Individual identification with organization, Other personal attributes.  *Domain:* Process; *Constructs*: Engagement, Opinion Leaders, Champions, Innovation Participants.  2.2. The analysis team was composed of the primary analyst and secondary analysts (AW, MK). All analysts independently coded each transcript using NVIVO12. The entire dataset was systematically coded line by line into constructs identified in step 2.1 where possible and additional codes were generated during the process to address the remaining concepts.  2.3. When an analyst independently identified a novel topic in a transcript, they would discuss if the topic fit into the one of the already identified constructs or a new construct needed to be generated.  *Additional themes generated included:* Huddle culture, Huddle outcomes, Huddle topics, Informal huddles, Intervention workload, Shift report, Sustainability.  2.4. The analysts met weekly to compare their coding, discuss discrepancies, and reach consensus on them. |
| 3. Memo creation | 3.1. One memo was created by RC (AW) for each timepoint that interviews were conducted.  *For instance, for site 1, two memos were created for pre- and post-implementation respectively. For analysis, quotes from both memos that were related similarly to the overall ratings were amalgamated. For site 2, one memo was created as interviews occurred throughout implementation, with no distinct timepoint.*  3.2. Memos were created using an adapted version of the CFIR memo template (<https://cfirguide.org/tools/tools-and-templates/>). Data was aggregated first by CFIR construct and aggregated within each construct by participant.  *For instance, in site 1’s pre-implementation memo, all comments coded as ‘Leadership engagement’ were grouped together, and then sorted and labelled by participant.* |
| 4. Construct ratings | 4.1. Constructs were rated using an adaptation of the CFIR rating rules (<https://cfirguide.org/tools/tools-and-templates/>). Ratings were comprised of valence (+/-) and strength (0, 1, 2). Asterisks (*) were used to indicate mixed findings in valence.  4.2. RCs assigned ratings to the same construct across all memos at one time, completing ratings for same construct across all sites and timepoints before moving on to the next construct.  *For instance,* *RCs assigned ratings to ‘Self-efficacy’ in site 1’s pre-implementation memo, site 1’s post-implementation memo, and site 2’s memo, before moving on to the next construct.*  4.3. Independently, RCs assigned ratings to each participant aggregated within a construct, basing the rating on their individual comments.  *For instance, participant 09 and 12 were coded for ‘Self-efficacy’ in site 2’s memo. Participants 09 and 12 were each assigned individual ratings, based on their respective comments. Participant 09 was assigned +1, and participant 12 was assigned -1 by AW.*  4.4. Independently, RCs assigned an overall rating for each construct, within each memo. Overall ratings were based on the individual participant ratings and were reflected the overall valence and strength of participant comments. Overall ratings that were mixed were marked as ‘X’  *For instance, based on participant 09 and 12’s ratings of +1 and -1, AW assigned an overall rating of 0 for ‘Self-efficacy’ in site 2’s memo.*  4.5. RCs met several times each week to compare individual and overall ratings and summaries, discuss discrepancies and come to a consensus on an overall rating for each construct, in each memo.  *For instance, RCs met to crosscheck their ratings and come to a consensus on 3 overall ratings for ‘Leadership engagement’.* |
| 5. Cross-comparison of sites | 5.1. Independently, members of the study team (AW, AK, MK, KM) performed a cross-comparison between sites of overall ratings assigned to each construct.  *Because implementation was terminated prematurely at site 2, timepoints were not synchronous between sites. Site 1’s post-implementation ratings were compared to site 2’s overall ratings.*  5.2. First, constructs were compared to identify those differentiating implementation between sites. Constructs were designation as ‘Strongly distinguishing’, ‘Weakly distinguishing’, and ‘Not distinguishing’ based on a construct’s dominance, i.e., those with the greatest discordance in valence and strength between sites, those with greatest reported frequency, and based on researchers’ judgement.  *For instance, ‘Communication’ was rated in site 1, post-implementation as +2, and in site 2 as -2. This construct was designation* *as ‘Strongly distinguishing’ between sites.*  5.3. Members met once to compare ‘Distinguishing factors’, discussing discrepancies, and coming to a consensus on the final designation for each construct.  *Unlike other published findings employing the CFIR, due to the shortened implementation process in site 2, a comparison between sites to elucidate true distinguishing factors leading to differences in success was not possible.*  5.4. Independently, members studied ‘Not distinguishing’ constructs for their level of influence on implementation. Not distinguishing constructs were further identified as ‘Strongly influential’, ‘Weakly influential’, and ‘Not influential’ based on strength of the sites’ ratings, and the nature of participants’ comments.  *For instance, ‘Knowledge and beliefs about the intervention’ was rated in site 1, post-implementation as +2, and in site 2 as +2. This was designated as ‘Strongly influential’ across both sites.*  5.5. Members met once to compare ‘Influential factors’, discussing discrepancies, and coming to a consensus on the final designation for each construct. |
